# From Qualitative to Quantitative AOP: A Case Study of Neurodegeneration

**DOI:** 10.3389/ftox.2022.838729

**Published:** 2022-03-30

**Authors:** Dennis Sinitsyn, Natàlia Garcia-Reyero, Karen H. Watanabe

**Affiliations:** ^1^ Arizona State University, School of Mathematical and Natural Sciences, Glendale, AZ, United States; ^2^ Oak Ridge Institute for Science and Education, Environmental Laboratory, US Army Engineer Research and Development Center, Vicksburg, MS, United States; ^3^ US Army Engineer Research and Development Center, Environmental Laboratory, Vicksburg, MS, United States

**Keywords:** qAOP, acetylcholinesterase inhibition, KER description, toxicity testing research needs, chemical risk assessment

## Abstract

Adverse outcome pathways (AOPs) include a sequence of events that connect a molecular-level initiating event with an adverse outcome at the cellular level for human health endpoints, or at the population level for ecological endpoints. When there is enough quantitative understanding of the relationships between key events in an AOP, a mathematical model may be developed to connect key events in a quantitative AOP (qAOP). Ideally, a qAOP will reduce the time and resources spent for chemical toxicity testing and risk assessment and enable the extrapolation of data collected at the molecular-level by *in vitro* assays, for example, to predict whether an adverse outcome may occur. Here, we review AOPs in the AOPWiki, an AOP repository, to determine best practices that would facilitate conversion from AOP to qAOP. Then, focusing on a particular case study, acetylcholinesterase inhibition leading to neurodegeneration, we describe specific methods and challenges. Examples of challenges include the availability and collection of quantitative data amenable to model development, the lack of studies that measure multiple key events, and model accessibility or transferability across platforms. We conclude with recommendations for improving key event and key event relationship descriptions in the AOPWiki that facilitate the transition of qualitative AOPs to qAOPs.

## Introduction

The ability to predict the potential hazard of chemicals is crucial to better understand and protect both human health and ecological receptors. Regardless of numerous international efforts to improve predictions, many challenges remain. The Adverse Outcome Pathway (AOP) framework (Ankley et al., 2010) is an increasingly accepted approach to link biological pathways at the molecular level to adverse outcomes. While the development of AOPs has increased substantially, the need for quantitative approaches using the AOP framework remains a challenge. It took years to develop what could be considered the first quantitative AOP (qAOP), and several approaches have been proposed to date (Conolly et al., 2017; Perkins et al., 2019b). The development of qAOPs is arguably one of the main challenges remaining within the AOP framework, nevertheless necessary in order to improve risk and hazard prediction.

The development of a qAOP logically follows AOP development given its function as a mathematical representation of the key event relationships (KERs) in an AOP. Different approaches have been used including: 1) fitting functions to key event (KE) data bounding a KER(s) (response-response method) (Doering et al., 2018; Doering et al., 2019; Zgheib et al., 2019; Song et al., 2020); 2) biologically based mathematical modeling using ordinary differential equations (aka systems biology modeling) (Muller et al., 2015; Gillies et al., 2016; Conolly et al., 2017; Zgheib et al., 2019); and recently 3) a causal modeling approach using a Bayesian Network (Jeong et al., 2018; Perkins et al., 2019a; Zgheib et al., 2019; Burgoon et al., 2020; Moe et al., 2021; Paini et al., 2022). Bayesian Networks, in particular, are useful for describing complex AOPs involving multiple pathways leading to an AO as long as there are no feedback loops. The KEs of the AOP can be taken as the nodes of the network and can even be used to model time dependencies in the form of Dynamic Bayesian Networks (Zgheib et al., 2019). Note that in this article, response-response relationships are defined as mathematical functions determined by a regression analysis, whereas in other publications, e.g., Paini et al. (2022), the response-response relationship is defined more broadly to include biologically based models that quantitatively relate two KEs. The merits and pitfalls of the response-response approach and biologically based modeling have been discussed (Schultz and Watanabe, 2018; Foran et al., 2019; Zgheib et al., 2019; Spinu et al., 2020), but a significant barrier to the development of qAOPs in any form is the availability of quantitative data amenable for mathematical model development.

The goal of this article is to improve the efficiency of converting a qualitative AOP into a qAOP. A workflow for qAOP development, electronic resources, and three case studies are described in Paini et al. (2022) based on a recent Lorentz workshop. In the following, challenges to qAOP development were identified by reviewing AOPs with WPHA/WNT^1^ endorsement by the Organisation for Economic Co-operation and Development (OECD, 2021) in the AOPWiki^2^, and through a case study on developing a qAOP for acetylcholinesterase (AChE) inhibition leading to neurodegeneration (Conrow et al., 2021). As the construction of AOPs are an ever-evolving process, and as we reviewed these AOPs in November 2021, it should be noted that the information contained in the list of endorsed AOPs and the information presented inside the AOPs may change over time, and what was available at the time of this review may not reflect what is available in the future. We selected AOPs with WPHA/WNT endorsement as it provided us with a relatively broad and manageable set of AOPs to review.

## Review of AOPs With OECD Status

We performed a review of AOPs with OECD status to determine how readily other KER descriptions would facilitate conversion from AOP to qAOP, and explore any similar challenges shared between AOPs. Determining confidence in an AOP and its associated KERs is established through weight of evidence (WoE) evaluations based on modified Bradford-Hill criteria involving biological plausibility, empirical support, and quantitative understanding (OECD, 2018). The process of determining confidence through said criteria has been discussed previously (Becker et al., 2015), and while confidence in the supporting data may be considered high for a qualitative AOP, the next step of converting to a qAOP requires a more specific, quantitative set of data. Our goal in this case was to review the cited quantitative data and categorize the AOPs based on how readily a qAOP could be developed based on the presentation of information and WoE for the quantitative understanding section. Our review of AOPs with OECD status included only those that provided a WoE evaluation for KERs (see Table 1).

**TABLE 1:** Categorization of AOPs with OECD Status based on presentation of quantitative data in the quantitative understanding section of the KER description. Total KERs include KERs between non-adjacent KEs. T = Written in text only with cited references, F = Includes figures extracted from articles, Ta = References are provided in a tabulated form. QU-WoE, Weight of Evidence under the quantitative understanding section.

AOP #	Title	QU-WoE for KERs in the AOP			Category
		Low	Moderate	High	
3	Inhibition of the mitochondrial complex I of nigro-striatal neurons leads to parkinsonian motor deficits	3	4	1	T, F, Ta
25	Aromatase inhibition leading to reproductive dysfunction	1	7	0	T
131	Aryl hydrocarbon receptor activation leading to uroporphyria	2	1	2	T, F
54	Inhibition of Na^+^/I^−^ symporter (NIS) leads to learning and memory impairment	10	3	2	T
23	Androgen receptor agonism leading to reproductive dysfunction (in repeat-spawning fish)	8	5	0	T
21	Aryl hydrocarbon receptor activation leading to early life stage mortality, *via* increased COX-2	1	4	0	T
150	Aryl hydrocarbon receptor activation leading to early life stage mortality, *via* reduced VEGF	4	3	0	T
42	Inhibition of thyroperoxidase and subsequent adverse neurodevelopmental outcomes in mammals	7	5	0	T
10	Binding to the picrotoxin site of ionotropic GABA receptors leading to epileptic seizures in adult brain	0	3	2	T
6	Antagonist binding to PPARα leading to body-weight loss	2	4	2	T

When quantitative data are available for a KER, a question arises as to how to go about extracting it for use in quantitative model development. Our review found that quantitative data are presented in a variety of ways, ranging from text with cited references to data presented in a tabulated form along with relevant figures. The majority of the AOPs reviewed currently contain text with cited references in the KER quantitative understanding section, although AOP 131 supplements some of the text with a figure (Farhat et al., 2021). In contrast, AOP 3 provides text and relevant figures for all KERs and includes tables of quantitative data (Bal-Price et al., 2019). It is important to note that while the presentation of data in the quantitative understanding section of an AOP varies depending on the AOP in question, it does not reflect an AOP’s capability to be converted to a qAOP. For example, AOP 25, Aromatase Inhibition Leading to Reproductive Dysfunction, has a qAOP while containing only text with cited references in the quantitative understanding sections (Villeneuve, 2021).

## Case Study: AChE Inhibition Leading to Neurodegeneration

**FIGURE 1:**
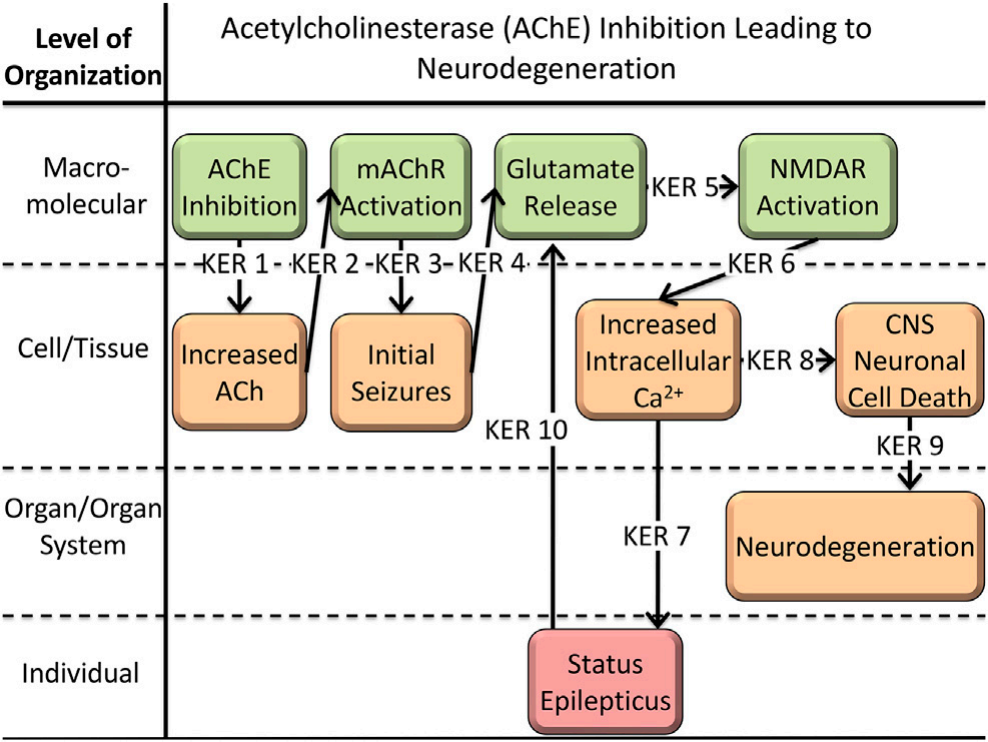
Graphical representation of AOP 281: AChE Inhibition Leading to Neurodegeneration (Conrow et al., 2021). Each arrow represents the key event relationship (KER) between key events (KE) of the AOP.

AChE inhibition leading to neurodegeneration is AOP 281 in the AOP Wiki (Conrow et al., 2021) and is currently under development (see Figure 1). The molecular initiating event (MIE) is AChE inhibition resulting in an excess of acetylcholine (ACh) in the synapse (KER 1). The build-up of ACh overactivates muscarinic acetylcholine receptors (mAChR) within the brain (KER 2), initializing local (focal) seizures (KER 3). Spreading of the focal seizure through glutamate release (KER 4) and subsequent activation of n-methyl-D-aspartate (NMDA) receptors (KER 5) propagates the excitotoxicity and leads to elevated intracellular calcium levels (KER 6), status epilepticus (KER 7), and ultimately cell death (KER 8) and neurodegeneration (KER 9). Additionally, status epilepticus induces further release of glutamate (KER 10), forming a positive feedback loop. The following text outlines our methods used during the conversion process, and the challenges we encountered.

### qAOP Development Methods

#### Literature Review

The first step in creating the qAOP was to examine the studies and data obtained during the construction of the qualitative AOP. We performed a comprehensive literature review that included the qualitative evidence previously obtained and examined additional studies found through publicly available databases, totaling over 200 papers examined. Concluding the review, we gathered and grouped the data into two categories: 1) model development, and 2) model evaluation. Ideally, model development data covers at least two adjacent key events, and if there is an abundance of data meeting this criterion then a dataset(s) could be set aside and used to evaluate the model’s predictive ability. In cases where data are reported for non-adjacent KEs, two outcomes are possible: 1) If the qAOP is being developed on response-response relationships and all KEs need to be in the qAOP, then these data should be used for evaluating the qAOP. 2) In a biologically based modeling context, data for non-adjacent KEs can be used for model development. However, as an example, given two datasets, one that contains data for three adjacent KEs, and another that only contains data for the first and third (non-adjacent) KEs, we would use the first data set for model development and the latter for model evaluation.

#### Quantitative Model Development

##### Data Needs

We initially planned to use a response-response relationship approach for the construction of a modular qAOP, however, while there were data available for KER 1, data to develop response-response relationships for the remaining KERs were not available. Thus, we chose a hybrid approach that would combine a response-response model for KER 1 with a biologically based model spanning KERs 2 through 10. Response-response relationships are built upon dose-response data for adjacent KEs, and linear regression analysis was used KER 1. For the remaining KERs, response-response data were sparse, and a feedback loop in the AOP (KERs 5, 6, 7, 10) precludes the use of response-response modeling and necessitates the development of a biologically based model. Of the papers reviewed for qAOP model development, approximately 10 studies in the primary literature resulted in useable quantitative data for model development (Michaels and Rothman, 1990; Kosasa et al., 1999; Kim et al., 2003; Falkenburger et al., 2010; Mergenthal et al., 2020), and model evaluation (Lallement et al., 1992; Marks et al., 1996; McDonough and Shih, 1997; Miller et al., 2015; Reddy et al., 2021).

Studies reporting data for non-adjacent KEs, while useful for model evaluation, are not ideal for developing quantitative models for KERs. For example, Miller et al. (2015) reported percent AChE inhibition (MIE) and hippocampal volume loss, but did not report data for any KEs adjacent to these endpoints. In AOP 281, hippocampal volume loss is a potential measure of the neurodegeneration adverse outcome. Though it may not be the best measure to use in a response-response relationship, a biologically based model could predict this endpoint among other indicators of neurodegeneration and utilize the study for model evaluation. Similarly, Lallement et al. (1992) reported measurements of ACh and glutamate. In this case, we could not determine a response-response relationship relating ACh concentration to glutamate. However, the data can be used to evaluate predictions from a biologically based model that incorporates mechanistic processes representing the intermediate KEs. We acknowledge that the published studies discussed were not designed with qAOP model development in mind, though they provide examples of where changes in experimental design and increased research funding could yield a more comprehensive understanding of underlying biological mechanisms.

Biological systems are often regulated by feedback loops, which requires development of a time-dependent biologically based model in contrast to a response-response relationship (Zgheib et al., 2019). At “steady-state”, when time dependencies are removed and derivatives with respect to time are set to zero, a response-response relationship could be used to relate the input into a feedback loop with the output, essentially ignoring the mathematical dynamics of the feedback loop (Zgheib et al., 2019). However, when a system is perturbed, and a feedback loop exists, a time-dependent biologically based model is needed to capture the system dynamics. Thus, biologically based models benefit from time series measurements of the associated KEs to identify model parameter values. In AOP 281, KERs 5, 6, 7, and 10 form a positive feedback loop requiring data to uniquely define model equations and parameter values. Response-response models work for linear pathways with one input and one output and are implemented sequentially through an AOP (Foran et al., 2019). In contrast, feedback loops involve more than one input/output for a KE (e.g., two inputs into the glutamate release KE and two outputs from the increased intracellular calcium KE), resulting in a non-linear pathway. As feedback loops are a commonly used regulatory mechanism in nature, methods to develop quantitative models should be encouraged instead of avoided.

In the context of high-throughput chemical toxicity applications, KE measurements need to be made quickly and inexpensively. Some studies report excellent data obtained through sophisticated measurement techniques that are not practical for use in chemical toxicity testing and risk assessment due to cost and time constraints. Such techniques might be described as part of the evidence in a KE description though the technique is impractical for measuring a KE in high-throughput toxicity testing. With respect to the status epilepticus KE, researchers used quantitative MRI to predict hippocampal damage based on changes in the structure and volume of the hippocampus after inducing status epilepticus through overactivation of mAChR by pilocarpine (Choy et al., 2010). While the data obtained are informative, the tools (i.e., the MRI) required are likely to be costly and impractical for toxicity testing applications. Ultimately, this issue can be applied more generally to the time and financial costs required of *in vivo* experiments compared to *in vitro*.

Distribution of available data throughout an AOP differs for KERs. In the context of AOP 281, KER 1 was supported by quantitative data that resulted in a response-response relationship. In contrast, data to develop a response-response relationship for KER 2 were not found. Similar data availability or lack thereof can be seen in many other AOPs. As an example, the quantitative understanding section of AOP 3 ranges from low to high depending on the KER in question (Bal-Price et al., 2019). This uneven distribution of data can be restrictive and prevent model developers from working with a single modeling approach to develop a qAOP. Thus, research funding that supports the collection of data for multiple (adjacent) endpoints in an AOP would facilitate qAOP model development tremendously.

##### Interspecies Differences in Biological Response

Consideration should also be given to interspecies differences in response to chemical stressors (Celander et al., 2011). Ideally, there should be equivalent measurable responses between the target species and the animal model(s) or *in vitro* assays that provide data. In the case of AOP 281, we started with rat data because there were significantly more studies available across the AOP than other animal models. By definition, AOPs are independent of chemical stressor, however data required to develop a qAOP are obtained from *in vivo* and/or *in vitro* experiments using chemical(s), and interspecies differences in the measured responses may occur. For example, in response to OPs, rats respond similarly to humans, though they have a 3-6 fold higher LD_50_ compared to humans when administered sarin intravenously, and guinea pigs have a 1.7-fold higher LD_50_ (Pereira et al., 2014). Pereira et al. attribute the fold difference in LD_50_ values to differences in OP metabolism between species, and quantitatively, this can be addressed through toxicokinetic modeling and methods to quantify measurement uncertainty and biological variability (Gelman et al., 1996; Bernillon and Bois, 2000; Jager, 2021). In terms of our qAOP, guinea pig data may be better suited for predicting human responses, but the data spanning the qAOP are insufficient. Thus, we will rely upon data from other species and use principles of interspecies extrapolation and allometric scaling (Davidson et al., 1986) as needed. For regulatory use, interspecies differences in biological responses could be quantified along with measurement uncertainty and biological variability using methods cited above.

Reducing the number of animals used in toxicity testing is a benefit of new approach methodologies such as *in vitro* assays and in silico models. Given the considerations above regarding interspecies differences, a wider array of *in vitro* assays focused on non-model organisms should be developed for ecotoxicology purposes (Hecker, 2018), and to provide a knowledge base that will improve our quantitative understanding of interspecies differences in biological response.

#### Reuse of Quantitative Models

Accessibility and transferability of established quantitative models are important factors to consider for accelerating qAOP development. Currently, there are two models that simulate cellular response to mAChR activation (Greget et al., 2016; Mergenthal et al., 2020), which could be extended for use in our qAOP. Mergenthal et al. (2020) describe a computational model of cholinergic modulation of CA1 pyramidal cells developed in the NEURON simulation environment, spanning KERs 2 to 3, while Greget et al. (2016) describes a simulation of a CA1 hippocampal cells responding to OP-induced neurotoxicity, spanning KERs 1 to 3. However, the model by Greget et al. was not accessible, and the NEURON simulation environment is too specialized for our qAOP. Thus, we will use Mergenthal et al. as a reference for KERs 2 and 3 to construct a biologically based model spanning KERs 2 to 10. The reuse of existing models can dramatically improve the pace of qAOP development, though access and cross-platform transferability are of concern. Additionally, models developed in proprietary or unfamiliar software can be restrictive to newer model developers. Tools built with user-friendly, open-source software and data exchange formats such as Systems Biology Markup Language (Hucka et al., 2003) are possible solutions.

## Discussion

Overall, the process of converting an AOP to a qAOP is time and resource-intensive and requires an abundance of quantitative data for the associated KERs. Some of the challenges presented above are not expected to be resolved for the foreseeable future. Costly measurements and the uneven distribution of data will remain an issue and will decrease in significance over time if and when new methods are developed or when research bridges the knowledge gaps in areas lacking in quantitative understanding. A recommendation for these challenges would be best aimed toward funding agencies placing additional funding into the areas identified by modelers to be lacking in data. Below are four additional recommendations pertaining to the remainder of the challenges.

Regarding the review of the AOPs with OECD status and their presentation of quantitative data, we recommend that quantitative data be presented in a more easily accessible form to facilitate use in a qAOP. Additionally, we would also like to emphasize the importance of quality data reporting. Data that are not produced under OECD guidelines still need to follow a standard to be considered reliable for regulatory applications. Hartung et al. (2019) provides the reader with existing reporting guidelines and discusses the need for and what constitutes Good *In Vitro* Reporting Standards (GIVReSt). The majority of quantitative data in KER descriptions are reported as in-text citations that requires a modeler to manually extract the data for qAOP development. This process could be shortened if the data were presented in a tabular form that combines data and quantitative relationships extracted from the cited sources. Factors that aide in this process include incorporating relevant figures from the cited studies with appropriate copyright permissions, tabulated data, and dose-response or response-response quantitative relationships. In the AOP Wiki, AOP 3 (Bal-Price et al., 2019) demonstrates these factors, as the KER description’s quantitative evidence section contains significant and detailed information. The individual KERs contain relevant figures and tabulated information of the studies supporting the AOP. Presentation of information in this manner required a significant amount of effort by the AOP authors, which will ultimately improve the rate at which an AOP can be converted to a qAOP.

Concerning the biological differences between species, we would like to highlight the need to understand the physiology of the organism to be used in modeling, and more specifically to know which chemical stressors can be used if multiple species are involved in model development. Using AOP 3 as an example, 1-methyl-4-phenyl-1,2,3,6-tetrahydropyridine (MPTP) is a compound commonly used in animal models of Parkinson’s disease. More specifically, the AOP 3 authors mention that the effect on mice produces Parkinsonian symptoms similar to that seen in humans, however rats are much less susceptible to MPTP, which would not be a good fit for a model (Petroske et al., 2001; Bal-Price et al., 2019). Understanding differences such as these are crucial to successful qAOP development. With the ultimate goal of 21st century toxicology moving away from *in-vivo* testing, greater emphasis should be placed on developing *in vitro* assays to be used as a replacement for animal studies (US EPA, 2021). In this case, research funding for the development of these assays and qAOPs would facilitate a move away from animal models by providing additional data sets for qAOP model development.

In the context of our case study, we would like to make recommendations for two of the challenges presented involving studies that measure multiple non-adjacent key events and model transferability. Currently, existing studies that measure non-adjacent key events are less ideal for modeling and better suited for evaluation. This highlights the need for studies that measure multiple adjacent key events. There exists a similar need for models following a biologically based approach or AOPs that contain feedback loops. Biologically based models benefit most from having both dose-response and time series data for multiple key events, and studies that can provide that set of data would be invaluable to qAOP model developers and would aide in the transition from AOP to qAOP. Lastly, in a recommendation aimed toward model developers, we suggest keeping model transferability in mind when developing a model, as this would simplify the process for both developers and data scientists looking to adapt available models for their needs. Hosting models in repositories such as Github or SourceForge enables version tracking and would benefit model development by allowing multiple authors to modify existing models to meet new needs. Additionally, during the construction of either a qualitative or qAOP, the authors may come across raw data or models that could support qAOP development. In this case, we suggest hosting additional materials in the AOP Wiki to allow for better data management and efficient model development. To that end, we would also like to recommend development of modular qAOP models for KERs that can be shared and re-used to fit a developer’s needs. Hosting of such models could take place in already existing repositories, such as BioModels (Malik-Sheriff et al., 2019).

In addition to their application in toxicity testing, AOPs and qAOPs have benefits beyond their original purpose. The design structure of AOPs can be helpful in other fields not associated with chemical risk assessment. The pharmacological and medical field could adapt the concept of AOPs to fit their needs. For example, recent efforts have begun in developing AOPs for COVID-19, known as the CIAO project^3^. Physicians could follow a similar modular approach in diagnosing and treating patients based on symptoms and treatment options. Additionally, AOPs can help identify knowledge gaps in a particular area (Leist et al., 2017). As AOPs are constructed from sources of published literature or sets of experimental data, their modular nature can easily highlight areas lacking in mechanistic understanding. Identification of these gaps will guide future studies and allow for a deeper understanding of the pathology in question (US EPA, 2020). Lastly, as progress continues in the development of AOPs and the addition of more qAOPs, the next logical step would be the integration of multiple qAOPs into qAOP networks. This might best be achieved with a Bayesian Network approach, as the structure of the AOPs and KEs naturally follow the form of the network (Perkins et al., 2019a). In conclusion, one may ask what specifically is needed to make these recommendations happen. Additional funding in the areas lacking in data suitable for model development would be a first major step, followed by a change in the culture of data sharing for better accessibility, and lastly, a change in best practices for how we write KE and KER descriptions. These changes will allow these recommendations come to fruition and will facilitate the transition from AOP to qAOP.

## Data Availability

The original contributions presented in the study are included in the article/supplementary material, further inquiries can be directed to the corresponding author.

## Footnotes

1 Working Party on Hazard Assessment/Working Group of the National Coordinators of the Test Guidelines Programme.

2
https://aopwiki.org/.

3
https://www.ciao-covid.net/.
